# Assessing Metabolic Ageing via DNA Methylation Surrogate Markers: A Multicohort Study in Britain, Ireland and the USA

**DOI:** 10.1111/acel.14484

**Published:** 2025-01-20

**Authors:** Kexin Xu, Belinda Hernández, Thalida Em Arpawong, Stephane Camuzeaux, Elena Chekmeneva, Eileen M. Crimmins, Paul Elliott, Giovani Fiorito, Beatriz Jiménez, Rose Anne Kenny, Cathal McCrory, Sinead McLoughlin, Rui Pinto, Caroline Sands, Paolo Vineis, Chung‐Ho E. Lau, Oliver Robinson

**Affiliations:** ^1^ MRC Centre for Environment and Health, Department of Epidemiology and Biostatistics School of Public Health, Imperial College London London UK; ^2^ MRC WIMM Centre of Computational Biology, Radcliffe Department of Medicine, Medical Sciences Division University of Oxford Oxford UK; ^3^ The Irish Longitudinal Study on Ageing (TILDA), Department of Medical Gerontology School of Medicine, Trinity College Dublin Dublin Ireland; ^4^ Leonard Davis School of Gerontology University of Southern California Los Angeles California USA; ^5^ National Phenome Centre and Imperial Clinical Phenotyping Centre, Section of Bioanalytical Chemistry, Department of Metabolism Digestion and Reproduction, IRDB Building, Imperial College London London UK; ^6^ NIHR Health Protection Research Unit in Chemical and Radiation Threats and Hazards London UK; ^7^ UK Dementia Research Institute at Imperial College London London UK; ^8^ Clinical Bioinformatics Unit IRCCS Istituto Giannina Gaslini Genoa Italy; ^9^ Ageing Epidemiology (AGE) Research Unit School of Public Health, Imperial College London London UK

**Keywords:** ageing, biological age, cohort studies, DNA methylation, epigenetics, metabolism, metabolomics, risk factors

## Abstract

Metabolomics and epigenomics have been used to develop ‘ageing clocks’ that assess biological age and identify ‘accelerated ageing’. While metabolites are subject to short‐term variation, DNA methylation (DNAm) may capture longer‐term metabolic changes. We aimed to develop a hybrid DNAm‐metabolic clock using DNAm as metabolite surrogates (‘DNAm‐metabolites’) for age prediction. Within the UK Airwave cohort (*n* = 820), we developed DNAm metabolites by regressing 594 metabolites on DNAm and selected 177 DNAm metabolites and 193 metabolites to construct ‘DNAm‐metabolic’ and ‘metabolic’ clocks. We evaluated clocks in their age prediction and association with noncommunicable disease risk factors. We additionally validated the DNAm‐metabolic clock for the prediction of age and health outcomes in The Irish Longitudinal Study of Ageing (TILDA, *n* = 488) and the Health and Retirement Study (HRS, *n* = 4018). Around 70% of DNAm metabolites showed significant metabolite correlations (Pearson's *r*: > 0.30, *p* < 10^−4^) in the Airwave test set and overall stronger age associations than metabolites. The DNAm‐metabolic clock was enriched for metabolic traits and was associated (*p* < 0.05) with male sex, heavy drinking, anxiety, depression and trauma. In TILDA and HRS, the DNAm‐metabolic clock predicted age (*r* = 0.73 and 0.69), disability and gait speed (*p* < 0.05). In HRS, it additionally predicted time to death, diabetes, cardiovascular disease, frailty and grip strength. DNAm metabolite surrogates may facilitate metabolic studies using only DNAm data. Clocks built from DNAm metabolites provided a novel approach to assess metabolic ageing, potentially enabling early detection of metabolic‐related diseases for personalised medicine.

## Graphical abstract

DNA methylation was used to develop surrogate markers for over 500 metabolites, which were subsequently combined to develop a novel hybrid clock to assess metabolic ageing. The clock gave strong predictions of age in three cohorts (British, Irish and American) and the derived age accelerations were associated with multiple ageing risk factors and health outcomes.

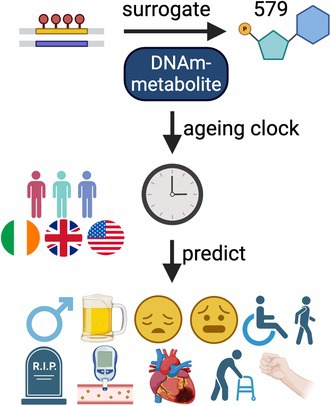

## 1 Introduction

With lifespan increasing faster than health span for the last two centuries, prolonging disease‐free years has become a focus of ageing research (Garmany, Yamada, and Terzic 2021). Ageing, a time‐dependent decline in physiological function and stress resistance, underlies most chronic diseases, including cancer, diabetes, neurodegeneration and cardiovascular disease (CVD) (Rutledge, Oh, and Wyss‐Coray 2022; López‐Otín et al. 2013). The rate of ageing varies among individuals due to factors like lifestyle, mental well‐being and socioeconomic factors (Abud et al. 2022). To quantify interindividual ageing variability, the concept of biological ageing was coined to denote the series of cellular and molecular changes preceding phenotypic and functional ageing (Ferrucci et al. 2018; López‐Otín et al. 2013). Hence, measuring biological age might allow early detection of individuals at risk of chronic diseases before symptoms appear (Ferrucci et al. 2018). Individuals with ‘accelerated ageing’ (i.e., higher biological age than chronological age) may be identified for lifestyle changes and potential early clinical interventions (Ferrucci et al. 2018).

To measure biological age, ‘omics’, the systematic analysis of sets of biomolecules such as epigenomics and metabolomics has been used to develop ‘ageing clocks’ in large cohorts (Rutledge, Oh, and Wyss‐Coray 2022). Based on age‐related methylation changes, the first‐generation DNA methylation (DNAm)‐based clocks, such as the blood‐based clock of Hannum et al. (2013) and the pan‐tissue clock of Horvath (2013), were trained on chronological age and have been shown to predict chronological age with extraordinary accuracy across multiple populations (Horvath and Raj 2018). Second‐generation clocks such as DNAm GrimAge (Lu et al. 2019) and PhenoAge (Levine et al. 2018) incorporated biomarker and mortality information into training to provide a more direct assessment of biological age. In parallel, several clocks have been developed on metabolomic data (Hertel et al. 2016; Robinson et al. 2020; Lau et al. 2024), which appear to provide complementary information to DNAm clocks (Jansen et al. 2021; Robinson et al. 2020). The metabolome has certain inherent advantages for assessing ageing, including the extensive overlap between ageing hallmarks and metabolism (López‐Otín et al. 2013; López‐Otín et al. 2016). As the final product of cellular metabolism, circulating metabolites can provide a summary of body‐wide biological processes associated with age (Robinson and Lau 2023). Despite the advantages of the metabolome, many metabolites are highly temporally variable (Bermingham et al. 2021), which may impair their utility to predict longer‐term sources of variation such as ageing. Also, metabolomic assessments across laboratories are generally unstandardised (Rutledge, Oh, and Wyss‐Coray 2022). In contrast, DNAm is inherently stable temporally (Gadd et al. 2022; Stevenson et al. 2020) and benefits from standardised measurements from commercial arrays (Shu et al. 2020). Hence, it has been applied to develop surrogate markers of circulating biomarkers, such as proteins (Gadd et al. 2022). A DNAm surrogate marker for the inflammatory marker, C‐reactive protein, showed greater temporal stability and a stronger association with age and cognitive decline than C‐reactive protein itself (Stevenson et al. 2020).

We hypothesised that DNAm surrogates for metabolites (‘DNAm‐metabolites’) may provide a more stable and longer‐term marker of metabolic ageing than metabolites alone. Therefore, we aimed to develop DNAm metabolites for around 600 serum metabolites measured by nuclear magnetic resonance (NMR) spectroscopy or ultrahigh‐performance liquid chromatography–mass spectrometry (UHPLC‐MS) platforms from over 800 participants within the UK Airwave Health Monitoring Study (Airwave). We compared associations with chronological age between the DNAm metabolites and the metabolites themselves. Next, we combined the DNAm metabolites to develop a DNAm‐metabolic clock and assessed its association with chronological age and risk factors of ageing. Finally, we assessed and validated the association of the DNAm‐metabolic clock with chronological age and a range of age‐related health outcomes in two independent cohorts, The Irish Longitudinal Study on Ageing (TILDA) cohort (*N* = 488) and the American Health and Retirement Study (HRS) cohort (*N* = 4018).

## 2 Results

### 2.1 Study Populations

**FIGURE 1:**
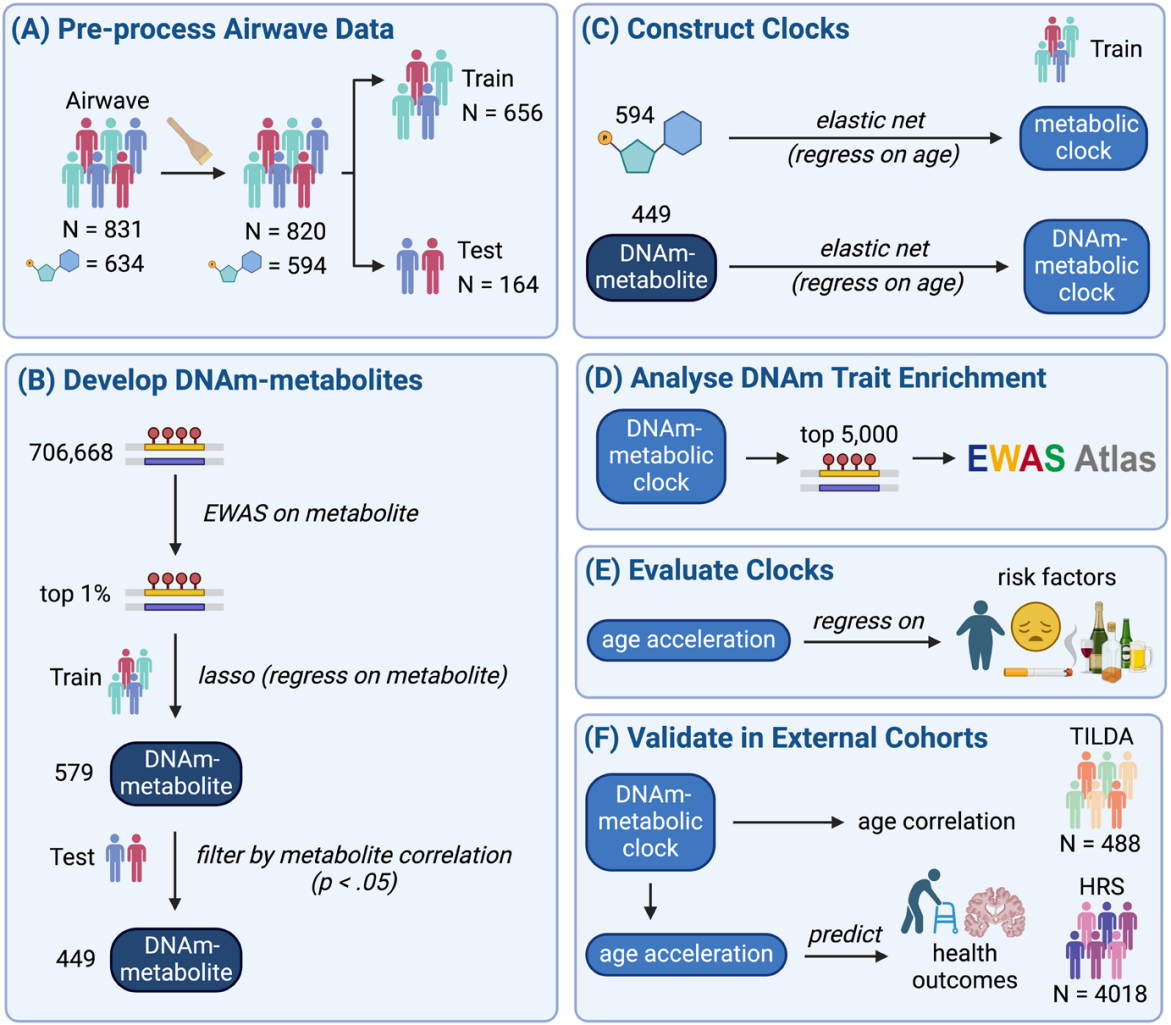
Workflow for the development and evaluation of DNAm‐metabolic and metabolic clocks. (A) The Airwave cohort (*N* = 831) was divided into training and test sets following data preparation. (B) Epigenome‐wide association studies (EWAS) were performed to select the 1% most significantly associated CpGs with each metabolite. Lasso regression of these CpGs with metabolites was used to develop 579 DNAm‐metabolite surrogates. DNAm metabolites were filtered by their correlation with metabolites in the test set (Bonferroni‐corrected *p* < 0.05). (C) Regression of chronological age on DNAm metabolites or metabolites was performed to develop DNAm‐metabolic and metabolic clocks. (D) Enrichment analysis was performed on the 5000 CpGs in the DNAm‐metabolic clock with the largest coefficients. (E) Age acceleration (residual from regression of predicted age on chronological age) was entered into a regression on noncommunicable disease risk factors. (F) The DNAm‐metabolic clock was assessed in two independent cohorts, The Irish Longitudinal Study on Ageing (TILDA) (*N* = 488) and the Health and Retirement Study (HRS) (*N* = 4018), in their ability to predict chronological age and health outcomes.

The overall study design is shown in Figure 1. After quality control (Figure 1A), the Airwave cohort included 820 participants with both DNAm and metabolomic data, aged 21–65 years, whose characteristics are summarised in Table 1. The demographics of this subset were similar to the full Airwave cohort (Elliott et al. 2014). As an occupational cohort, Airwave is representative of employees of the UK police force but not the general UK population due to the generally better health and preponderance of males (58.2%) and white British ethnicity (97.6%) (Elliott et al. 2014).

**TABLE 1:** Characteristics of 820 participants in the Airwave cohort. Details on each category are in the Method section.

Characteristic	Category	*N* (%)
Age (years), mean (SD)		41.5 (9.2)
Sex	Female	343 (41.8%)
	Male	477 (58.2%)
Ethnicity	White	800 (97.6%)
	Nonwhite	20 (2.4%)
BMI	Normal	266 (32.4%)
	Overweight	388 (47.3%)
	Obese	166 (20.2%)
Alcohol intake	None	58 (7.1%)
	Moderate	705 (86.1%)
	Heavy	56 (6.8%)
Smoking	Nonsmoker	543 (66.2%)
	Former smoker	194 (23.7%)
	Current smoker	83 (10.1%)
Physical activity	High	479 (58.4%)
	Moderate	211 (25.7%)
	Low	130 (15.9%)
Diabetes	No	675 (82.4%)
	Prediabetic	110 (13.4%)
	Diabetic	34 (4.2%)
Hypertension	No	547 (66.8%)
	Yes	272 (33.2%)
Trauma	No	691 (84.3%)
	Yes, without posttraumatic stress disorder (PTSD)	102 (12.4%)
	Yes, with PTSD	27 (3.3%)
Anxiety	No	619 (77.3%)
	Borderline	109 (13.6%)
	Yes	73 (9.1%)
Depression	No	549 (67%)
	Minimal symptoms	201 (24.5%)
	Yes	69 (8.4%)

### 2.2 DNAm Metabolites Were Associated With Chronological Age and Predicted Chronological Age Closely

**FIGURE 2:**
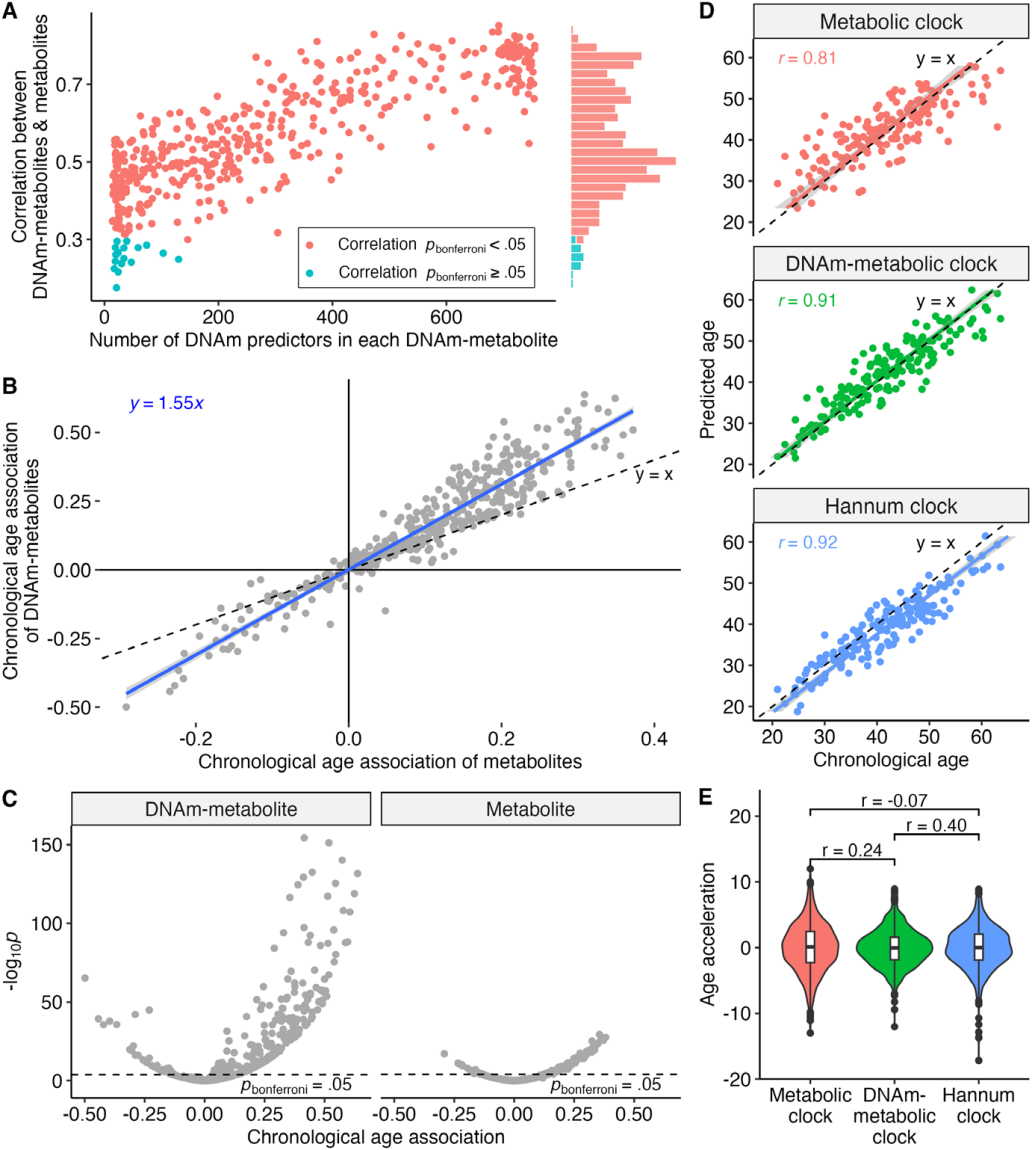
DNAm metabolites predicted chronological age closely. (A) Each metabolite was regressed on DNAm (DNA methylation) in the training set (*N* = 656) to build DNAm metabolites. DNAm metabolites were assessed for Pearson's correlation (*r*) with their corresponding metabolites in the test set (*N* = 164). (B, C) DNAm metabolites generally had stronger chronological age associations in (B) magnitude and (C) significance than metabolites. Chronological age associations were derived by sex‐adjusted regression of DNAm metabolites or metabolites on age (*N* = 820) and expressed as changes in the standard deviation of metabolites or DNA metabolites per year change of age. (D) Regression of age on 594 metabolites or 449 DNAm metabolites was performed in the training set using elastic net regularisation to select 193 metabolites or 177 DNAm metabolites to build metabolic and DNAm‐metabolic clocks. The clocks were compared to the Hannum clock in their chronological age correlation in the test set. (E) Age acceleration was derived as the residual from the regression of predicted age on chronological age (N = 820).

Using a linear combination of CpGs via least absolute shrinkage and selection operator (lasso) regression in a randomly selected training set (80% of Airwave, *n* = 656), DNAm‐metabolite models were successfully constructed for 579 of 594 metabolites, including lipids, lipoproteins and small molecules (Table S1). DNAm metabolites were assessed by their correlation with the original metabolites in the test set (remaining 20% of Airwave, *n* = 164) (Figure 1B, and Table S1). A total of 113 DNAm metabolites showing a bimodal distribution were removed as they were likely confounded by single nucleotide polymorphisms (Petersen et al. 2014). Further analysis focused on the 449 DNAm metabolites with significant (Bonferroni‐corrected *p* < 0.05, nominal *p* < 10^−4^) correlations with their respective metabolites in the test set (*r*: mean = 0.58, range: [0.30, 0.85]) (Figure 2A). DNAm metabolites that included more CpG predictors generally had higher correlations with metabolites (Figure 2A). On average, 301 CpGs (range: [14, 755]) were used to construct these DNAm metabolites. Correlations between metabolites and DNAm metabolites were very similar upon further adjustment of blood cell type proportions (Figure S1).

Associations with chronological age were assessed for both DNAm metabolites and metabolites through linear regression (individual coefficients in Table S1). Most DNAm metabolites had stronger age associations than their corresponding metabolites in magnitude (Figure 2B) and significance (Figure 2C). On average, DNAm metabolites were 1.6 times more strongly associated with chronological age than the corresponding metabolites (Figure 2B). A higher proportion of DNAm metabolites (63.3%) were significantly (Bonferroni‐corrected *p* < 0.05) associated with chronological age than metabolites (42.6%) (Figure 2C).

To compare DNAm metabolites and metabolites in age prediction, they were combined into multivariable predictors of chronological age using elastic net regularisation in the training set (Figure 1C). A total of 177 DNAm metabolites and 193 metabolites were, respectively, selected by elastic net regression to build the DNAm‐metabolic and metabolic clocks. The clocks were compared to the Hannum clock, a blood‐based DNAm clock similarly trained on chronological age (Hannum et al. 2013). The DNAm‐metabolic clock predicted chronological age (*r* = 0.91) in the test set as closely as the Hannum clock (*r* = 0.92), outperforming the metabolic clock (*r* = 0.81) (Figure 2D). Age acceleration was calculated as the residual from the regression of predicted age on chronological age, with a positive value indicating accelerated ageing. While metabolic and Hannum age accelerations were uncorrelated (*r* = −0.05, *p* = 0.28) with each other, DNAm‐metabolic age acceleration was correlated with both metabolic (*r* = 0.27, *p* < 0.001) and Hannum age accelerations (*r* = 0.36, *p* < 0.001) (Figure 2E).

### 2.3 CpG Sites in DNAm‐Metabolic Clock Were Enriched in Multiple Metabolic Features

To see whether the CpG sites included in the DNAm‐metabolic clock were enriched for sites related to metabolic factors, we performed enrichment analyses using the EWAS catalogue on the 5000 CpGs with the largest absolute coefficients in the DNAm‐metabolic clock, and for comparison, the 71 CpGs in the Hannum clock (Figure 1E).

**FIGURE 3:**
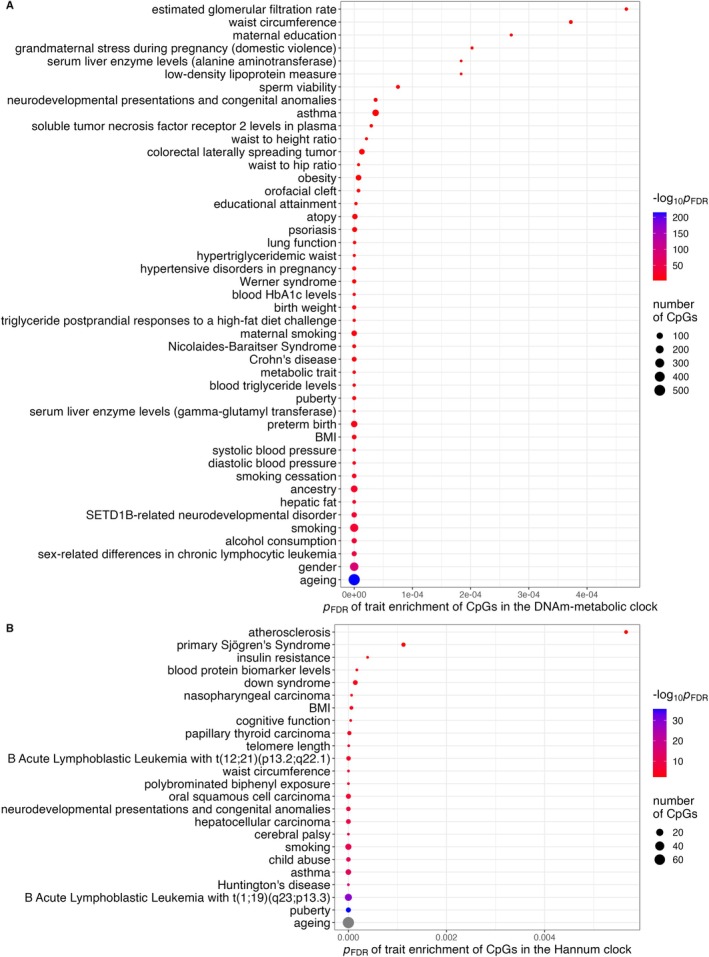
Trait enrichments of (A) the top 5000 CpGs in the DNAm‐metabolic clock (top 45 traits shown) and (B) the 71 CpGs in the Hannum clock. FDR‐adjusted *p* showed the probability of CpGs co‐occurring with trait‐related CpGs in EWAS Atlas.

CpGs in Hannum and DNAm‐metabolic clocks had, respectively, 24 and 71 trait enrichments (FDR‐adjusted *p* < 0.05) (Figure 3). A total of 552 (11%) DNAm‐metabolic CpGs and 71 (100%) Hannum CpGs were enriched in ageing. At least 19 traits of DNAm‐metabolic CpGs had metabolic links, including alcohol consumption, hepatic fat, diastolic and systolic blood pressure, BMI, serum liver enzyme levels, blood triglyceride levels, metabolic trait, Crohn disease, dietary triglyceride response, blood HbA1c levels, hypertensive disorders in pregnancy, hypertriglyceridemic waist, obesity, waist‐to‐hip/height ratios, low‐density lipoprotein measure and waist circumference (Figure 3A). In contrast, only two traits of Hannum CpGs had clear metabolic links, that is, waist circumference and BMI (Figure 3B).

### 2.4 DNAm‐Metabolic Age Acceleration Captured Many Metabolic Clock Risk Factors

**FIGURE 4:**
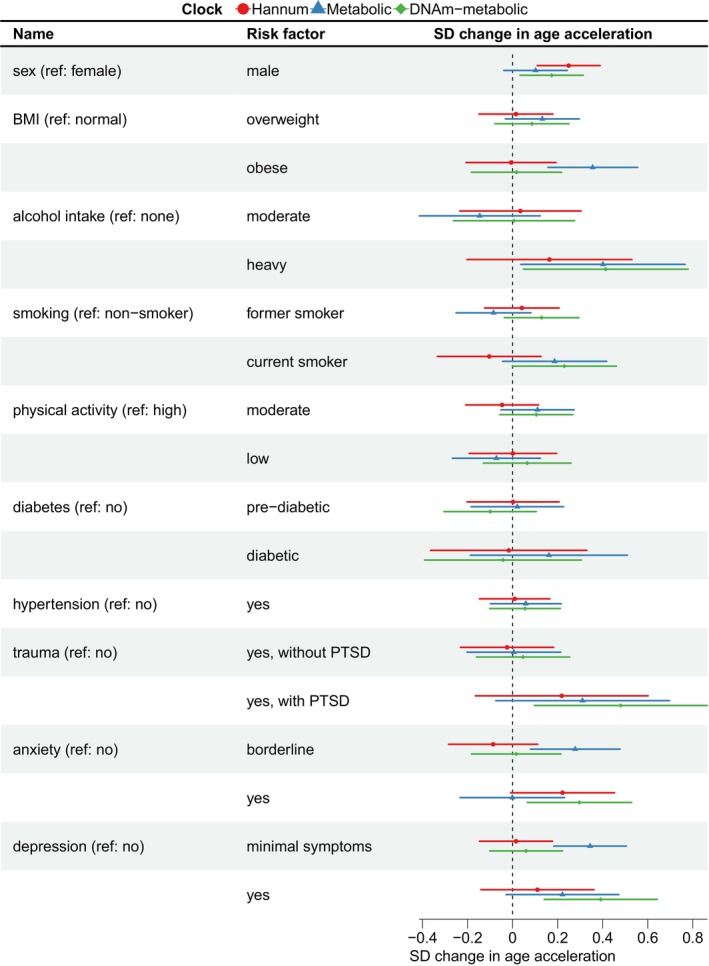
DNAm‐metabolic and metabolic age accelerations shared associations with many noncommunicable disease risk factors. Age accelerations were scaled and linearly regressed on each risk factor (effect sizes shown with 95% confidence intervals), adjusting for age, sex and ethnicity (*N* = 820).

To assess whether DNAm‐metabolic age acceleration could capture noncommunicable disease risk factors, we performed regressions of age accelerations on each risk factor and adjusted for age, sex and ethnicity (*N* = 820) (Figure 1D). Metabolic age acceleration was associated (*p* < 0.05) with obesity (0.36 SD, 95% CIs [0.16, 0.56]), heavy drinking (0.40 SD, 95% CIs [0.04, 0.77]), borderline anxiety (0.28 SD, 95% CIs [0.08, 0.48]) and depressive symptoms (0.34 SD, 95% CIs [0.18, 0.51]). DNAm‐metabolic age acceleration was associated with male sex (0.17 SD, 95% CIs [0.03, 0.31]), heavy drinking (0.41 SD, 95% CIs [0.05, 0.78]), recent trauma with PTSD (0.48 SD, 95% CIs [0.10, 0.86]), diagnosed anxiety (0.30 SD, 95% CIs [0.07, 0.53]) and diagnosed depression (0.39 SD, 95% CIs [0.14, 0.64]). Hannum age acceleration was only associated with male sex (0.25 SD, 95% CIs [0.11, 0.39]) (Figure 4). In short, DNAm‐metabolic age acceleration captured all but one risk factor (i.e., heavy drinking, anxiety, depression, but not obesity) associated with metabolic age acceleration, while having additional associations with male sex and recent trauma with PTSD. Additional adjustments for BMI and smoking status did not markedly change the result (Figure S2).

## 3 Independent Assessment of DNAm‐Metabolic Clock in Two External Cohorts

We applied the DNAm‐metabolic clock to DNAm data in TILDA and HRS (Figure 1F). We could not validate the metabolic clock externally due to the lack of a matching metabolite dataset. The TILDA study is a nationally representative survey of people over age 50 in the Republic of Ireland (McCrory et al. 2021). The subset with DNAm data included 488 participants (50.4% male) aged 50–87 years (sample characteristics described in Table S2). The HRS is a nationally representative longitudinal survey of people aged over 50 years in the USA (Sonnega et al. 2014). The subset with DNAm data included 4018 participants (41.5% male) aged 50–100 years and was ethnically more diverse than Airwave and TILDA (sample characteristics described in Table S3).

**FIGURE 5:**
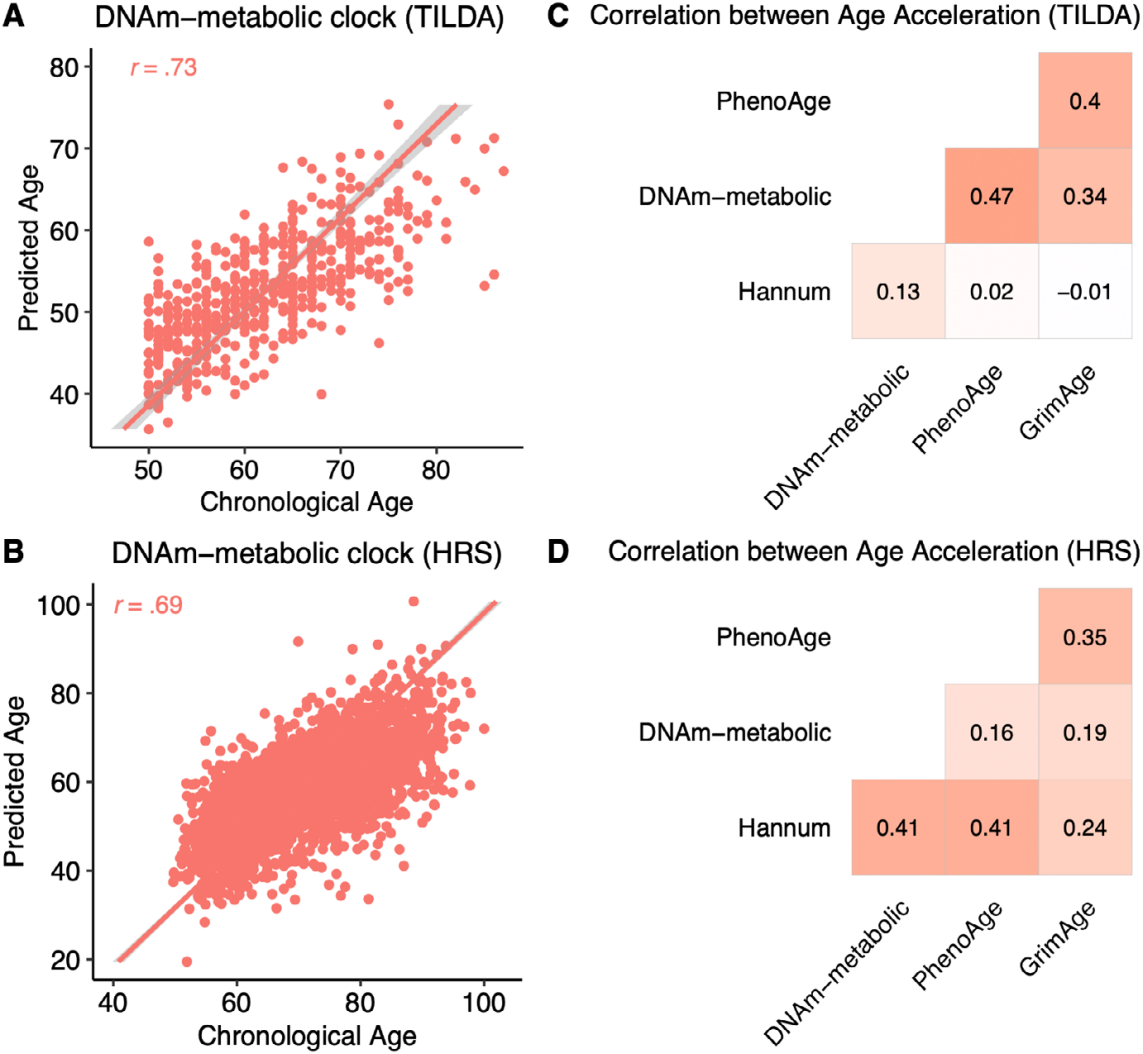
Validation of the DNAm‐metabolic clock in TILDA (*N* = 488) and HRS (*N* = 4018). (A, B) The DNAm‐metabolic clock predicted chronological age with a high Pearson's correlation in both cohorts. (C, D) Age acceleration from the DNAm‐metabolic clock showed weak‐to‐moderate correlations (0.13–0.47) with age accelerations from other clocks in both cohorts.

The DNAm‐metabolic clock predicted chronological age with a strong correlation in both cohorts (*r* = 0.73 in TILDA and 0.69 in HRS, Figure 5A,B). Age acceleration was derived from the DNAm‐metabolic clock and compared to three published epigenetic clocks (Hannum clock, PhenoAge and GrimAge). Hannum clock was similarly trained on chronological age like the DNAm‐metabolic clock, while PhenoAge and GrimAge are ‘second‐generation’ clocks trained on time to mortality (Lu et al. 2019; Levine et al. 2018). GrimAge, in particular, has been shown in TILDA to outperform other epigenetic clocks in predicting age‐related phenotypes and outcomes (McCrory et al. 2021). In TILDA, age acceleration from the DNAm‐metabolic clock showed moderate correlations with those from PhenoAge and GrimAge (*r* = 0.34 and 0.47) and a weak correlation with Hannum age acceleration (*r* = 0.13) (Figure 5C). In HRS, DNAm‐metabolic age acceleration showed a moderate correlation with Hannum age acceleration (*r* = 0.41) and weak correlations with those from PhenoAge and GrimAge (*r* = 0.16 and 0.19) (Figure 5D).

**FIGURE 6:**
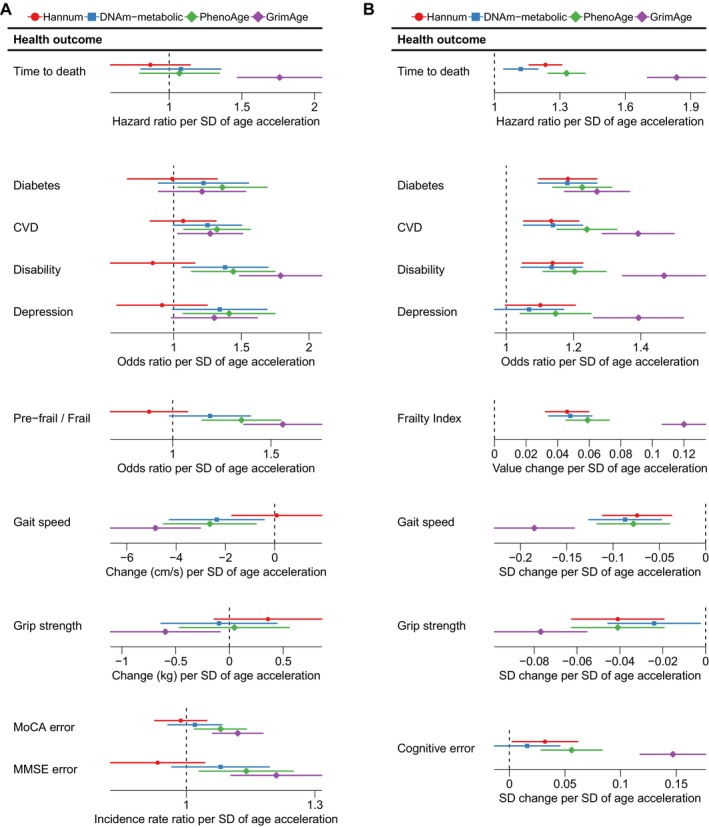
Age acceleration from the DNAm‐metabolic clock predicted multiple health outcomes in TILDA (*N* = 488) and HRS (*N* = 4018). (A) Health outcomes were regressed on scaled age accelerations in TILDA (effect sizes shown with 95% confidence intervals), adjusting for age and sex, using Cox regression (time to death), logistic regression (cross‐sectional diabetes, cardiovascular disease [CVD], disability, depression and prefrailty/frailty), linear regression (gait speed and grip strength) and negative binomial regression (errors in the Montreal Cognitive Assessment [MoCA] and the Mini‐Mental State Examination [MMSE]). (B) Health outcomes were regressed on scaled age accelerations in HRS, adjusting for age, sex and ethnicity, using Cox regression (time to death), logistic regression (diabetes, CVD, disability and depression) and linear regression (frailty index, gait speed, grip strength and cognitive error).

We tested the associations of age accelerations against incident all‐cause mortality (12‐year mortality in TILDA with 52 deaths and 6‐year mortality in HRS with 736 deaths), ageing‐related disease and disability (diabetes, CVD, disability, frailty and depression), physical function (gait speed and grip strength) and cognitive function (errors in the Montreal cognitive assessment (MoCA) and the mini‐mental state examination (MMSE) in TILDA and the telephone interview for cognitive status in HRS). Age accelerations from all clocks were associated with more health outcomes in HRS than in TILDA, likely due to the larger sample size in HRS (Figure 6).

In TILDA, the DNAm‐metabolic clock outperformed the Hannum clock in all health outcome associations. More specifically, while the Hannum clock did not predict any health outcome, the DNAm‐metabolic clock predicted gait speed (−2.35 cm/s in gait speed per SD increase in age acceleration, 95% CIs [−4.28, −0.42]) and disability (odds ratio per SD increase in age acceleration: 1.38, 95% CIs [1.01, 1.90]). Additionally, while the Hannum clock very often showed unexpected directions of effect in TILDA, the DNAm‐metabolic clock showed expected directions of effect with all health outcomes, including marginal associations with CVD (odds ratio: 1.25, 95% CIs [0.97, 1.61]) and depression (odds ratio: 1.34, 95% CIs [0.95, 1.9]) (Figure 6A). The second‐generation clocks (GrimAge and PhenoAge) each predicted eight health outcomes (*p* < 0.05), outperforming the Hannum and DNAm‐metabolic clocks. Additional adjustments for BMI and smoking status weakened health outcome associations for all clocks; nevertheless, the DNAm‐metabolic clock retained the expected directions of effect (Figure S3).

In HRS, the DNAm‐metabolic clock predicted seven of nine health outcomes (*p* < 0.05), except depression and cognitive error, where the expected directions of association were shown. The DNAm‐metabolic age acceleration was associated with time to death (hazard ratio per SD increase in age acceleration: 1.12, 95% CIs [1.05, 1.20]), diabetes (odds ratio: 1.18, 95% CIs [1.10, 1.27]), CVD (odds ratio: 1.14, 95% CIs [1.06, 1.22]), disability (odds ratio: 1.14, 95% CIs [1.05, 1.23]), frailty (0.05 increase in frailty index, 95% CIs [0.04, 0.06]), gait speed (−0.09 SD, 95% CIs [−0.13, −0.05]) and grip strength (−0.02 SD, 95% CIs [−0.04, −0.00]). Its performance was generally on par with the Hannum clock, showing similar associations except slightly weaker although still significant associations with time to death and grip strength. Compared to PhenoAge, the DNAm‐metabolic clock showed similar strengths of prediction for gait speed, while having slightly weaker but significant (*p* < 0.05) predictions for diabetes, CVD, disability, frailty and grip strength. GrimAge outperformed all other clocks (Figure 6B). Additional adjustments for BMI and smoking status did not markedly change the result (Figure S3).

Overall, the DNAm‐metabolic clock consistently predicted chronological age in TILDA and HRS. The derived age acceleration significantly (*p* < 0.05) predicted disability and gait speed in TILDA and showed marginal associations with CVD and depression. In HRS, the DNAm‐metabolic age acceleration significantly (*p* < 0.05) predicted time to death, diabetes, CVD, disability, frailty, gait speed and grip strength. While the DNAm‐metabolic clock had weaker predictions of health outcomes than GrimAge and sometimes PhenoAge, it markedly outperformed the Hannum clock in the Irish TILDA cohort and showed broadly similar performance as the Hannum clock in the more ethnically diverse HRS cohort.

## 4 Discussion

We demonstrated the use of DNAm as surrogates for over 400 metabolites in the Airwave cohort. The surrogates, DNAm metabolites, showed stronger associations with chronological age than the experimentally measured metabolites, suggesting they may better capture long‐term metabolic changes associated with ageing. We developed the DNAm‐metabolic clock based on these surrogates. The clock gave an improved prediction of chronological age than a metabolic clock constructed directly from metabolomic data. CpGs used in the DNAm‐metabolic clock were enriched for many metabolic traits. Moreover, compared to the metabolic clock or the established Hannum clock, age acceleration from the DNAm‐metabolic clock was associated with more noncommunicable disease risk factors, including heavy drinking, anxiety, depression, male sex and recent trauma with PTSD. When externally tested in the Irish TILDA cohort and the more ethnically diverse HRS cohort, the DNAm‐metabolic clock predicted chronological age with a strong correlation and the derived age acceleration was significantly associated with ageing outcomes like disability and gait speed in both cohorts and additionally, time to death, diabetes, CVD, frailty and grip strength in HRS.

Using DNAm to develop metabolite surrogates offers several advantages. We found that DNAm‐metabolites had on average 60% greater magnitude of association with chronological age than the original metabolites. As DNAm levels are comparatively more stable markers, DNAm‐metabolite surrogates may be interpreted as providing cumulative, longer‐term markers of metabolic phenotypes, which may be less sensitive to short‐term fluctuations caused by recent diet and lifestyle (López‐Otín et al. 2016; Gadd et al. 2022; Stevenson et al. 2020). For modelling a long‐term process such as ageing, DNAm‐metabolite surrogates may reduce noise due to short‐term variation and better capture the metabolic changes associated with ageing. Furthermore, using DNAm as metabolite surrogates may facilitate metabolic studies (not limited to age prediction) in cohorts with only DNAm data. The availability of more standardised DNAm arrays might make models based on DNAm metabolites more reproducible than those based on metabolites. Finally, relative metabolome coverage is currently limited compared to genome coverage by DNAm array, presenting challenges to metabolic pathway analyses. With DNAm‐metabolite surrogates, biological pathway investigations might alternatively be performed with DNAm enrichment analysis. Two other recent studies have explored the DNAm surrogate approach for modelling metabolite levels. In one study, DNAm was used as surrogates for 64 ^1^H‐NMR metabolic features (Bizzarri et al. 2024), finding that 23 surrogates (36%) had mean *r* > 0.35 with the corresponding metabolites in fivefold cross‐validation test sets. In another study of 299 LC–MS metabolites (Chen et al. 2023), 266 surrogates (89%) showed significant (*p* < 0.05) correlations with metabolites, with mean *r* = 0.49 in the training set. In comparison, our study presents the largest range of metabolites yet to be modelled with DNAm. Furthermore, our surrogates showed overall improved correlations with metabolites, which might be attributed to differences in metabolomic assays between studies, processing, predictor selection and the multivariable modelling algorithm used.

In some respects, the near‐perfect prediction of chronological age by first‐generation epigenetic clocks such as Hannum limited their relevance for assessment of biological age. CpG sites selected in first‐generation clocks are by design less affected by environmental factors; the derived age acceleration is less variable, limiting statistical power. Furthermore, the underlying biology mainly relates to epigenetic maintenance (Horvath and Raj 2018) rather than assessing broader biological ageing processes. Second‐generation epigenetic clocks such as GrimAge introduced a two‐step modelling process incorporating DNAm surrogates to increase their relevance to biological ageing. Our DNAm‐metabolic clock represents a hybrid between the pure DNAm clocks (e.g., Hannum) and metabolic clocks, as judged by the correlation between the clock‐derived age accelerations in Airwave. The relevance of the DNAm‐metabolic clock to metabolic ageing processes was supported by the enrichment of the included CpGs in many ageing‐related metabolic traits. For example, enrichments in blood triglyceride levels, postprandial triglyceride responses and hypertriglyceridemic waist correspond with the role of triglyceride as an ageing marker (Han et al. 2017). Enrichments in low‐density lipoprotein could reflect age‐related cholesterol changes (Schubert et al. 2006). Other ageing‐related enrichments included waist circumference (Stevens, Katz, and Huxley 2010), waist‐to‐hip/height ratios (Glei et al. 2011; Stevens, Katz, and Huxley 2010), blood pressure (Glei et al. 2011), blood HbA1c level (Dubowitz et al. 2014), liver fat (Ogrodnik et al. 2017) and markers of heavy drinking (serum levels of gamma‐glutamyl transferase (Kim, Kisseleva, and Brenner 2015) and alanine aminotransferase (Dong et al. 2010)). In contrast, the Hannum clock had very few metabolic enrichments.

Despite being trained on working‐age adults of predominantly white British in Airwave, our DNAm‐metabolic clock transferred well to the Irish TILDA cohort of older individuals and the American HRS cohort of older and more ethnically diverse individuals, demonstrating the broader utility of the DNAm surrogate approach. In TILDA, the clock gave a good prediction of chronological age and health outcomes including disability and gait speed, markedly outperforming the Hannum clock similarly trained on chronological age. Generally, in TILDA, effect sizes of DNAm‐metabolic age acceleration with health outcomes were similar to those for PhenoAge, except for weaker associations with cognition. In HRS, the DNAm‐metabolic clock prediction performance was strong for chronological age and seven of nine health outcomes, namely, time to death (from 6‐year mortality data), diabetes, CVD, disability, frailty, gait speed and grip strength. In HRS, where we observed a greater correlation between Hannum and DNAm‐metabolic age accelerations, associations with health outcomes were more similar among PhenoAge, Hannum and DNAm‐metabolic clocks. Since the DNAm‐metabolic clock generally had similar effect sizes in health outcome predictions between TILDA and HRS, this difference in relative performance appears to be due to weaker observed associations with the Hannum clock and PhenoAge in TILDA. The weakened associations may be due to the more ethnically and/or geographically distinct training populations used for the Hannum clock and PhenoAge (Hannum et al. 2013; Lu et al. 2019) relative to the TILDA population. GrimAge showed the strongest prediction of health outcomes across TILDA and HRS.

GrimAge is considered a ‘second‐generation’ clock as it was trained directly on mortality like PhenoAge, in addition to incorporating circulating biomarker data and smoking information. We lacked sufficient longitudinal mortality data in the subsample of Airwave with DNAm data to train on mortality. Although second‐generation clocks are more sensitive to ageing risk factors and showed improved predictions of age‐related diseases generally (Lu et al. 2019), they will to a greater extent capture extrinsic contributions to ageing, such as early effects of diseases, than intrinsic metabolic ageing effects. While the DNAm‐metabolic clock may be considered ‘first‐generation’ as it was trained on chronological age, it is also part of a new generation of clocks termed ‘Generation Explainable’ (Sehgal et al. 2024) that aims to improve the interpretation of clocks by incorporating information from genetic markers (Ying et al. 2024), physiological system assessments (Sehgal et al. 2023) or other omics markers (Chen et al. 2023). Epigenetic clock development is a field undergoing rapid development and validation (Moqri et al. 2024). The DNAm‐metabolic clock contributes to these efforts by proposing an approach based on targeting markers directly related to metabolism. As such, the DNAm‐metabolic clock might be more suited as an endpoint in intervention studies focused on improving metabolic health via, for example, diet and exercise. Changes in individual DNAm metabolites could be measured for further biological interpretability.

Limitations of this study include the younger age range and low ethnic diversity of participants in Airwave. While the younger age range might limit the predictive performance of the clock of the chronological age in older individuals, it allowed the clock to assess age acceleration in working‐age adults, when many metabolic diseases originate. Another limitation of the study is the use of cross‐sectional data for training that may be more susceptible to confounding by cohort effects than longitudinal repeat surveys. Secondly, DNAm metabolites and the DNAm‐metabolic clock were developed using linear regression that assumed linearity and additivity among predictors; however, age has nonlinear associations with some CpGs (Bernabeu et al. 2023) and might be affected by CpG‐CpG interactions (de Lima Camillo, Lapierre, and Singh 2022). Hence, nonlinear machine learning models might enhance prediction, if not interpretability, evidenced by the improved age prediction over linear models shown by a DNAm clock based on neural networks (de Lima Camillo, Lapierre, and Singh 2022) and metabolic clocks using multivariate adaptive regression splines (Lau et al. 2024).

Nonetheless, the strengths of our study include the external validation assessment of the DNAm‐metabolic clock in TILDA and HRS and the broad range of risk factors and health outcomes explored, including validated measures of psychological factors such as trauma, anxiety and depression. The study also benefitted from incorporating NMR and UHPLC‐MS platforms and the comprehensive metabolite annotation from raw metabolic features to capture a broad spectrum of metabolites.

In general, biological clocks could provide additional information to clinical markers and potentially facilitate early detection of diseases years before onset (Ferrucci et al. 2018). Compared to individual disease risk factors, biological age is a health score that integrates multidimensional risks and thus might have more versatile applications (Marengoni et al. 2011). The utility of biological age markers for health evaluation was demonstrated in an immunorestoration trial using GrimAge to measure epigenetic ageing reversal (Fahy et al. 2019), and a stem cell reprogramming study using several epigenetics clocks to measure cell rejuvenation (Olova et al. 2019).

In summary, we have demonstrated the use of DNAm to develop surrogate markers for a broad range of metabolites. The resulting DNAm metabolites had stronger chronological age associations than metabolites, suggesting they could provide a more stable assessment of metabolic ageing. We have presented a DNAm‐metabolic clock that was enriched for metabolic traits and outperformed the metabolic‐only and the DNAm‐only Hannum clocks in sensitivity to ageing risk factors in Airwave. In the external testing cohort TILDA, the DNAm‐metabolic clock predicted chronological age with a strong correlation; the derived age acceleration was associated with disability and gait speed, markedly outperforming the Hannum clock. In the larger and ethnically more diverse testing cohort HRS, the DNAm‐metabolic clock predicted chronological age and seven health outcomes including time to death. The contribution of this study is multifold. The DNAm‐metabolite surrogates can allow future metabolic studies when only DNAm data are available, provide longer‐term markers of metabolic phenotypes and offer a more standardised measurement than metabolome profiling. The clock built from DNAm metabolites provided a hybrid between a metabolic and a DNAm clock and offered a novel way to predict metabolic ageing from DNAm. As age‐related morbidities have gained prevalence, identifying those with accelerated ageing and targeting them with behavioural changes or therapeutic interventions might ultimately slow ageing and prolong health span (Bürkle et al. 2015). Our DNAm‐metabolic clock might offer such a way to enable early detection of metabolic‐related diseases for personalised medicine.

## 5 Methods

### 5.1 Airwave Study Population and Covariates

DNAm metabolites and the clocks were developed within a subset (*N* = 831) of Airwave, an occupational cohort study of participants from 28 British police forces (Elliott et al. 2014) recruited from 2006 to 2015. Ethical approval was obtained from the National Health Service Multi‐Site Research Ethics Committee (MREC/13/NW/0588).

The study measured sociodemographic, clinical, lifestyle and psychological data. Covariates were classified as follows. Ethnicity was classified as ‘white’ and ‘non‐white’. BMI was split into normal, overweight and obese with cut‐offs at 25 and 30 kg/m^2^. Alcohol intake was classified as ‘none’, ‘moderate’ (≤ 14 units/week for women and ≤ 21 units/week for men) and ‘heavy’ (> 14 units/week for women and > 21 units/week for men). Participants were categorised into ‘non‐smoker’, ‘former smoker’ and ‘current smoker’. Physical activity was determined as ‘high’, ‘moderate’ or ‘low’ by the International Physical Activity Questionnaire (The IPAQ group 2016). Participants were labelled as ‘pre‐diabetic’ if they had HbA1c ≥ 4.8% and < 6.5% and ‘diabetic’ if they had HbA1c ≥ 6.5% or a diabetes diagnosis. Participants were classified as hypertensive if they had a diagnosis, treatment, a systolic blood pressure ≥ 140 mmHg or a diastolic blood pressure ≥ 90 mmHg. Trauma was classified as ‘no’, ‘yes, without PTSD’ or ‘yes, with PTSD’ depending on whether the participant had a work‐related traumatic incident in the last 6 months and developed PTSD as assessed using the Trauma Screen Questionnaire (Brewin et al. 2002). Anxiety was classified as ‘no’, ‘borderline’ or ‘yes’ using the Hospital Anxiety and Depression Scale (Zigmond and Snaith 1983). Depression was categorised into ‘no’, ‘minimal symptoms’ and ‘yes’ using the Patient Health Questionnaire‐9 (Kroenke, Spitzer, and Williams 2001).

Missing covariates were imputed using the mice ‘Multivariate Imputation by Chained Equations’ algorithm (van Buuren and Groothuis‐Oudshoorn 2011). Besides covariates presented above, other variables were included for more accurate imputation (age, sex, study centre, rank, marital status, education and time since last eaten).

### 5.2 Airwave DNAm

A total of 930 Airwave participants had 834,012 CpG sites measured by Infinium MethylationEPIC BeadChip in DNA extracted from plasma samples. Data acquisition and preprocessing have been described elsewhere (McCartney et al. 2021). A total of 127,344 CpG sites (15.3%) with > 1% missing rate were excluded. The rest underwent k‐nearest‐neighbour imputation using the impute package (Hastie et al. 2022). As recommended for differential methylation analysis, DNAm *β*‐values were transformed into *M*‐values followed by mean‐centring and standard scaling using the formula (Du et al. 2010).

$$M=\log_{2}\frac{\beta }{1-\beta }$$

### 5.3 Airwave Metabolomics

Untargeted metabolomics at the National Phenome Centre was measured by NMR and UHPLC‐MS for 2618 participants, of which 820 had DNAm data. Details on data acquisition and preprocessing are available elsewhere (Robinson et al. 2020; Lewis et al. 2022). UHPLC‐MS untargeted metabolic profiles were annotated using the peakPantheR package (Wolfer et al. 2021) and NMR metabolic profiles were annotated by the commercial Bruker in vitro diagnostics platform (Bruker Biospin, Germany) (Jiménez et al. 2018) to identify a total of 634 named metabolites, including small molecules (e.g., amino acids), lipoproteins, fatty acids and lipids. Details on metabolite measurement and annotation are provided in the Supporting Information.

A total of 30 metabolites were excluded, including 22 metabolites with > 20% missing or zero values and eight drug‐linked metabolites (i.e., acetaminophen, caffeine, cotinine, ethanol, metformin, nicotine, quinine and warfarin) that may confound risk factor associations of the clocks. Ten metabolites were measured by both NMR and UHPLC‐MS, so the measurement with the weaker age association was removed for better age prediction. A total of 594 (93.7%) metabolites were included in subsequent analyses.

Next, 11 participants with > 5% missing metabolites were excluded. Since NMR has lower sensitivity than UHPLC‐MS (Emwas 2015), left‐censored quantile regression imputation was performed on NMR metabolites using imputeLCMD to impute the metabolites missing by being below the detection limit (Lazar and Burger 2022) and k‐nearest‐neighbour imputation was performed on UHPLC‐MS metabolites using impute (Hastie et al. 2022). Metabolites were natural log transformed to mitigate skewness, mean‐centred and scaled to unit variance.

### 5.4 DNAm‐Metabolite Construction

DNAm metabolites were developed for each metabolite (Figure 1B). Participants were divided into training and test sets (4:1). To develop DNAm metabolites within the training set (*N* = 656), linear regression was performed for each metabolite on its top 1% most significantly associated (i.e., lowest *p*‐values) CpGs, using lasso regularisation in glmnet to reduce the number of predictors (Tibshirani 1996; Friedman, Hastie, and Tibshirani 2010). Tenfold cross‐validation was used to finetune the regularisation strength.

To mitigate the confounding effects of SNPs on CpG‐metabolite associations (Petersen et al. 2014), DNAm‐metabolites showing bimodal distributions were removed using ‘is.bimodal’ in the LaplacesDemon package and ‘modetest’ in the multimode package, followed by visual inspection (Hall, Hall, and Statisticat 2021; Ameijeiras‐Alonso, Crujeiras, and Rodríguez‐Casal 2021). Next, DNAm metabolites showing significant (Bonferroni‐corrected *p* < 0.05). Pearson's correlation with the original metabolites in the test set were selected for subsequent analyses.

### 5.5 Age Association Evaluation

To determine chronological age associations, metabolites or DNAm metabolites underwent individual regression on chronological age in multiple sex‐adjusted linear models using ‘mlm’ from omics, followed by Bonferroni correction (Campanella 2016).

### 5.6 Ageing Clock Construction

We developed metabolic and DNAm‐metabolic clocks from linear regression of chronological age on metabolites or DNAm metabolites in the training set with elastic net regularisation in glmnet (Friedman, Hastie, and Tibshirani 2010; Zou and Hastie 2005) and tenfold cross‐validation to finetune the regularisation parameters. Elastic net was used instead of lasso as the numbers of predictors were similar to the number of participants. The Hannum clock was developed from 71 CpGs using methyclock (Pelegi‐Siso et al. 2021).

### 5.7 DNAm Enrichment Analysis

To compare biological traits captured by DNAm‐metabolic and Hannum clocks, we performed enrichment analyses using the EWAS Toolkit (Xiong et al. 2022) on the 5000 CpGs with the biggest absolute coefficient size in the DNAm‐metabolic clock and the 71 CpGs in the Hannum clock. Weighted Fisher's exact test was used to calculate the probability of these CpGs co‐occurring with trait‐related CpGs in the EWAS Atlas (Xiong et al. 2022).

### 5.8 Age Acceleration Calculation and Risk Factor Evaluation in Airwave

Age accelerations were calculated as residuals from regressions of clock‐predicted ages on chronological age and scaled to unit variance (Jansen et al. 2021). To derive risk factor associations of each clock, we performed linear regressions of age accelerations on each risk factor of premature mortality (sex, BMI, smoking, alcohol intake, physical activity, hypertension, diabetes, depression, trauma and anxiety) and adjusted for age, sex and ethnicity. BMI and smoking were additionally adjusted for as a sensitivity analysis. In this exploratory analysis, we presented associations with risk factors associated at the nominal *p* < 0.05 significance level.

### 5.9 TILDA Study Population and Covariates

We assessed the DNAm‐metabolic clock among 488 participants from TILDA, a prospective population‐representative study of adults aged ≥ 50 years in Ireland with surveys of 2‐year intervals from 2009 (Kearney et al. 2011; Donoghue et al. 2018). Ethical approval was obtained from the Trinity College Dublin Faculty of Health Sciences Research Ethics Committee. Data were collected on sociodemographic, cognitive ability, lifestyle and physical and mental health. Details on the preprocessing of epigenetic data and health outcomes are in the Supporting Information.

### 5.10 HRS Study Population and Covariates

We also assessed the DNAm‐metabolic clock among 4018 participants from HRS, a nationally representative longitudinal survey of adults aged over 50 years in the USA (Sonnega et al. 2014). Ethical approval was obtained from the University of Michigan Institutional Review Board. Data were collected on sociodemographic, cognitive ability, lifestyle and physical and mental health. Details on the preprocessing of epigenetic data and health outcomes are in the Supporting Information.

### 5.11 Assessment of the DNAm‐Metabolic Clock in TILDA and HRS Cohorts

We assessed the DNAm‐metabolic clock among 488 TILDA participants and 4018 HRS participants for its association with chronological age and risk factors. To build the clock, we applied the beta coefficients from the DNA‐metabolic clock derived in Airwave to methylation data in TILDA and HRS measured by Infinium MethylationEPIC BeadChip. Missing CpGs from TILDA and HRS (around 10% and 1% due to quality control) were imputed using median values of the corresponding CpGs from Airwave. We calculated age acceleration similarly to Airwave for the DNAm‐metabolic clock, and for comparison, the Hannum clock, PhenoAge and GrimAge. In TILDA, we performed regression of each health outcome on scaled age accelerations using the Cox proportional hazards model (time to death), logistic regression (cross‐sectional diabetes, CVD, disability, prefrailty/frailty and depression), linear regression (gait speed and grip strength) and negative binomial regression (due to overdispersion in MoCA and MMSE errors). In HRS, we performed the regression of each health outcome on scaled age accelerations using the Cox proportional hazards model (time to death), logistic regression (cross‐sectional diabetes, CVD, disability and depression) and linear regression (frailty index, gait speed, grip strength and cognitive error). The regression was adjusted for age and sex in both cohorts and additionally adjusted for ethnicity in HRS, as the TILDA cohort is ubiquitously white Irish and the HRS cohort is more ethnically diverse. BMI and smoking were additionally adjusted for as a sensitivity analysis.

## Author Contributions

O.R. and C.‐H.E.L. conceived, designed and supervised this study. K.X. conducted most analyses, wrote the first draft of the manuscript and revised subsequent drafts. B.H. performed the analyses in the TILDA cohort. T.E.A. performed the analyses in the HRS cohort. P.E. supervised data collection in Airwave. R.A.K., C.M. and S.M. supervised data collection in the TILDA cohort. E.M.C. supervised data development in HRS. G.F. processed the epigenetic data. R.P., B.J., E.M.C., C.S. and S.C. acquired and processed the metabolomic data. P.V. acquired the funding for the epigenetic data. All authors reviewed, edited and approved the final manuscript.

## Conflicts of Interest

The authors declare no conflicts of interest.

## Supporting information


Data S1.

## Data Availability

The data supporting the findings of this study are available upon application to the Data Access Committee for the Airwave cohort (https://police‐health.org.uk/applying‐access‐resource). TILDA data are deposited in the Irish Social Sciences Data Archive (ISSDA) https://www.ucd.ie/issda/data/tilda/. Access to the TILDA raw DNAm data is facilitated through a secure hot desk system at their premises in Trinity College Dublin https://tilda.tcd.ie/data/accessing‐data/hotdesk/. HRS data are available from the National Institute on Aging Genetics of Alzheimer's Disease Data Storage Site (NIAGADS) (NG00153) with an approved HRS data NIAGADS data access request (DAR). Information on how to apply for access can be found on the NIAGADS website. Code, coefficients and instructions for building the DNAm‐metabolic clock and the constituent 177 DNAm‐metabolites are available at https://github.com/Kexin‐xu‐01/DNAm‐metabolic‐age/. Details on each DNAm metabolite are available in Table S1.
