# Supplementary material for: Assessing Metabolic Ageing via DNA Methylation Surrogate Markers: A Multicohort Study in Britain, Ireland and the USA
Source: Aging Cell. 2025 Jan 20;24(5):e14484. doi: 10.1111/acel.14484 (PMC12073893; doi:10.1111/acel.14484)
Supplement: Supplementary file 1 — Data S1. [file ACEL-24-e14484-s001.zip › DNA metabolic age SI.docx]

**Supplementary Information**

***NMR Metabolic Profiling Analysis of Human Blood Plasma Samples in Airwave***

Plasma samples were analysed as previously described (Dona et al. 2014). Sample analysis was undertaken in a run order unrelated to key study questions/cohort demographics. In brief, plasma samples were centrifuged at 12000 g at 4°C for 5 min, and subsequently, 350 μL of supernatant were added into 96-well plates. Additional volume from each sample was used to prepare an internal quality control (QC) pool. Two internal and two external QC samples were added to each plate and analysed together with the study samples. Plasma sample plates were positioned into a 215 Gilson Liquid Handler robot and 300 μL of plasma were loaded into 4’’ 5 mm NMR tubes and mixed with 300 μL of H_2_O:D_2_O buffer. Spectra were acquired on a Bruker Advance III 600 MHz spectrometer operating at 14.1 T and equipped with a 5 mm BBI probe with a ^2^H-decoupling probe including a z-axis gradient coil. A refrigerated Sample Jet robot was used to store samples before and after acquisition at 5 °C and to transfer samples into the spectrometer for measurement. Acquisition temperature was calibrated to 310 ± 0.1 K using a chemical thermometer. Instrument performance was assessed regularly using standard samples. Quantitation values were obtained by analysing an ad-hoc standard sample. For each plasma sample, three NMR experiments were acquired in automation; a general profile ^1^H NMR water pre-saturation experiment using a one-dimensional pulse sequence where the mixing time of the 1D-NOESY experiment is used to introduce a second pre-saturation time (32 scans, 98,304 data points, spectral width of 30 ppm), a 2D J-resolved experiment (2 scans and 40 increments), and a spin echo edited experiment using the Carr-Purcell-Meiboom-Gill pulse sequence which filters out signals from fast T2 relaxing protons from molecules with slow rotational correlation times such as proteins and other macromolecules (32 scans, 73,728 data points, spectral width of 20 ppm). Free induction decays of all 1D spectra were multiplied by an exponential function equivalent to 0.3 Hz line-broadening before applying the Fourier transform. All Fourier-transformed spectra were automatically corrected for phase and baseline distortions and referenced to the glucose doublet at 5.233 ppm. 2D J-resolved spectra were also tilted and symmetrised after transformation. NMR spectra were subsequently quality-controlled using the nPyc toolbox (Sands et al. 2019). 41 metabolites and 112 lipoproteins were quantified for targeted NMR analyses using the *in vitro* diagnostics platform from Bruker Biospin (Jiménez et al. 2018).

***UHPLC-MS Metabolic Profiling Analysis of Human Blood Plasma Samples in Airwave***

Plasma samples were prepared and run by UHPLC-MS as previously published (Izzi-Engbeaya et al. 2018; Lewis et al. 2022). Samples were subjected to reversed-phase chromatography (RPC) tailored for complex lipid separation and hydrophilic interaction liquid chromatography (HILIC) for small polar metabolite separation. For QC assessment and data pre-processing, a QC sample was initially prepared by pooling equal parts of each study sample. A dilution series was created from the pooled QC sample (10x 100%, 5x 80%, 3x 60%, 3x 40%, 5x 20%, 10x 1%). In brief, 50 μL aliquots were taken from each sample and the pooled QC and diluted 1:1 v/v with ultrapure water. Protein was removed by adding organic solvent (diluted sample/isopropanol in 1:4 v/v ratio for RPC and diluted sample/acetonitrile in 1:3 v/v ratio for HILIC). Mixtures of method-specific authentic reference materials and internal standards were added (at the dilution stage for HILIC and protein precipitation stage for RPC) to monitor data quality during acquisition. All analyses were performed on ACQUITY UPLC instruments (Waters Corp., Milford, MA, USA) coupled to Xevo G2‐S TOF mass spectrometers (Waters Corp., Manchester, UK) via a Z‐spray electrospray ionisation source operating in both positive and negative ion modes to produce lipid positive and negative datasets (LPOS and LNEG respectively) and a HILIC positive dataset (HPOS). The pooled QC sample was acquired every ten study samples throughout the analysis and a dilution series sample set was acquired at the beginning and end of the run. Raw data was converted to the mzML open-source format and signals below an absolute intensity threshold of 100 counts were removed using the MSConvert tool in ProteoWizard (Chambers et al. 2012). Feature extraction was performed in XCMS (Smith et al. 2006) and the elimination of potential run-order effects and feature filtering was performed using the nPYc-Toolbox (Sands et al. 2019). Only features measured to high precision (relative standard deviation, RSD, in pooled QC samples < 30%) and high linearity in response (correlation to dilution factor in dilution series > 0.7) were retained and put forward for biological analysis.

***Targeted Metabolite Extraction for Airwave***

Feature extraction was performed using *peakPantheR* (Wolfer et al. 2021), an R package for targeted integration of chemical signals from liquid chromatography-mass spectrometry datasets. Briefly, empirical retention time and theoretical m/z values from an in-house database of metabolite/lipid annotations were used to integrate endogenous metabolites/lipids.

## ***TILDA Epigenetic Data Pre-processing***

Epigenetic data in TILDA underwent pre-processing and QC. Ten samples were excluded, of which five had sex discordance, four were duplications, and one failed the Illumina bisulphite conversion QC check. 116,154 probes were deemed unreliable and excluded due to having detection *p* > 0.01 and/or < 4 hybridising beads in > 5% of the samples, leaving a total of 749,705 for analysis. Normal-exponential out-of-band normalisation was performed in the remaining 488 samples.

***HRS Epigenetic Data Pre-processing***

DNA methylation was measured using DNA extracted from the buffy coat using the Infinium Methylation EPIC BeadChip by the Advanced Research and Diagnostics Laboratory at the University of Minnesota.  DNA samples were randomised across analytic plates by age, cohort, sex, education, and ethnicity along with 39 pairs of blinded duplicates. Analysis of duplicate samples showed a correlation above .97 across all CpG sites. Data preprocessing and quality control were performed using the *minfi* package in R (Aryee et al. 2014).  A total of 3.4% of the methylation probes were removed from the final data as their detection *p*-value fell below .01 (*n* = 29,431 out of 866,091).  Analysis for failed samples was done after removing detection *p*-value failed probes. A total of 58 samples were removed after applying a 5% cutoff. Sex-mismatched samples and any controls (cell lines and blinded duplicates) were excluded from the analyses. The final analytic data set includes 97.9% of the originally plated samples. Missing beta methylation values were imputed with the mean beta methylation value of the given probe across all samples (Faul et al. 2023).

***TILDA Health Outcomes***

Mortality status and cause of death were determined by inspecting death certificates over a 12-year follow-up until 31st January 2022 using data linkage between TILDA and the General Register Office in Ireland (Ward et al. 2020).

Diabetes was characterised by self-reported diagnosis and/or use of any of the following anti-diabetic medications as per the WHO Anatomical Therapeutic Classification system codes: insulins and analogues (A10A), blood-glucose-lowering drugs excluding insulins (A10B) or other diabetes drugs (A10X).

Cardiovascular disease was measured as participants self-reporting a lifetime history of any of the following: angina, heart attack, heart failure, stroke, transient ischaemic attack, or heart murmur.

## Disability was measured using the sum of the total number of self-reported limitations from the Activities of Daily Living (Katz et al. 1970) and the Instruments of Daily Living (Graf 2008) scales. Activities of Daily Living include difficulties with (1) dressing, (2) walking across a room, (3) bathing or showering, (4) eating, such as cutting up food, (5) getting in or out of bed, and (6) using the toilet. Instruments of Daily Living include (1) preparing a hot meal, (2) doing household chores, (3) shopping for groceries, (4) making telephone calls, (5) taking medications, and (6) managing money.

Depression was defined as the use of antidepressant medication ATC N06A and/or a score of 16 or above on the long form of the Centre for Epidemiologic Studies Depression Scale (Radloff 1977).

Frailty was defined using the 32-item frailty index (FI) which captures information regarding disability, symptoms, signs, and diseases affecting various organ systems (Roe et al. 2017). The FI has previously been operationalised in TILDA and scales the number of deficits on a scale of 0-1. Participants are then characterised as robust (FI < 0.1), pre-frail (0.1 ≤ FI < 0.25) or frail (FI ≥ 0.25) based on their index scores (Roe et al. 2017; Romero-Ortuno 2013).

Gait speed was assessed using a 4.88 m computerised walkway with embedded pressure sensors (GAITRite, CIR Systems Inc., New York, NY). The mean gait speed was taken across 2 walks along the mat at their normal walking speed and measured in cm/s. Each walk started 2.5 m before and ended 2 m after the walkway so that readings were not affected by acceleration and deceleration.

Grip strength was measured using a Baseline Hydraulic Hand Dynamometer. Respondents were instructed to hold the device in their hand with the forearm at a right angle to their upper arm and squeeze as hard as they could. This procedure was repeated twice on each hand and the mean value to the nearest kg was reported across all four attempts.

Global cognitive functioning was assessed using the Mini-Mental State Examination (Folstein et al. 1975) and the Montreal Cognitive Assessment (Nasreddine et al. 2005). Both result in a score between 0-30. The number of errors was modelled by subtracting the number of incorrect responses from the total score of 30.

***HRS Health Outcomes***

Time to death was coded in months from the date when blood samples were collected for DNA methylation measures to the follow-up period, up to the 2022 survey date. Respondents were coded as 0 for still living or as 1 for 736 individuals who died during the follow-up period, with the mean number of months = 62.9 (*SD* = 18.0).

Diabetes was characterised by self-reported diagnosis.

Cardiovascular disease was characterised by the self-reported lifetime history of any of the following: heart attack, coronary heart disease, angina, congestive heart failure, stroke, transient ischemic attack, or other heart problems

## Disability was measured using the sum of the total number of self-reported limitations from the Activities of Daily Living (Katz et al. 1970) and the Instruments of Daily Living (Graf 2008) scales.

Depression was assessed using the 8-item Center for Epidemiologic Studies Depression Scale (CESD). Respondents were asked to report yes (coded as 1) or no (coded as 0) to eight questions. The responses are then summed (range 0-8) with a cutoff of four or more depressive symptoms used to reflect a level of symptomatology that is clinically important but does not indicate a diagnosis (Measures & Arbor 2008; Zivin et al. 2010).

The Frailty Index was developed by including measures used in the HRS sister study, the English Longitudinal Study of Ageing (ELSA) (Mekli et al. 2018). The HRS Frailty Index consists of 39 measures: difficulty with activities of daily living (walking, transferring, bathing, dressing, eating), instrumental activities of daily living (making a phone call, managing money, taking medications, shopping, preparing meals), memory (immediate and delayed word recall), psychological problems, joint replacement, difficulty with vision or hearing, difficulty with Nagi functioning (using a map, yard work, walking one block, sitting, getting up from chair, climbing several flights of stairs without resting, climbing one flight of stairs without resting, stooping/kneeling/crouching, reaching arms above shoulder level, pushing or pulling large objects, picking up a dime, lifting or carrying weights over 10 pounds, self-reported health, and the presence of chronic diseases (hypertension, angina, congestive heart failure, diabetes, stroke, lung disease, arthritis, osteoporosis, cancer, heart attack). Binary variables were coded as 0 or 1 to indicate the absence or presence of deficits. Continuous and ordinal variables were categorised from 0 to 1 to indicate various degrees of deficits. The sum of deficit scores was then divided by the number of total measures and multiplied by 100.

Gait speed was assessed using a 98.5-inch (250-cm) course in the home of the respondent, created with a tape measure and masking tape marking the starting and ending points. Respondents were asked to complete two timed walks. Walking speed was conducted with respondents aged 65 years or above in HRS. The average of two-timed walks was calculated.

Grip strength was assessed using a Smedley spring-type hand dynamometer. Participants were instructed to squeeze the device as hard as they could while standing with their arms at their sides and with the elbow flexed at a 90-degree angle. After one practice trial, two measurements were taken for each hand, alternating hands. The maximum reading from the four trials was used to indicate grip strength capacity.

The cognitive error score indicates the number of errors made on the modified Telephone Interview of Cognitive Status Exam (mTICS) (Fong et al. 2009). The exam included assessments of immediate recall, delayed recall, serial 7s, and backwards counting tasks, with a score range of 0-27. For immediate recall, interviewers read a list of 10 words, and participants were asked to recall as many as possible (range 0-10). For delayed recall, participants were asked to recall the same 10 words after about 5 minutes (range 0-10). For the serial 7s task, participants were asked to count backwards from 100 by 7 for 5 trials (e.g., 93, 86, 79, 72, 65; range 0-5). For the backwards counting task, participants were asked to count backwards from 20 by 1 for 10 continuous numbers, scoring 0 errors if they did it successfully on two trials, 1 error if they did it successfully on one trial, and 2 errors if successful on neither trial.

**Supplementary Table 2. TILDA Cohort Characteristics.**

| **Characteristic** | **Mean (*SD*) or N (%)** |
| --- | --- |
| Age | 62.2 (8.35) |
| Sex Female | 242 (49.6%) |
| Education |  |
| Primary | 113 (23.2%) |
| Secondary | 167 (34.2%) |
| Third | 208 (42.6%) |
| BMI | 28.8 (5.03) |
| Smoking History |  |
| Never | 191 (39.1%) |
| Former | 212 (43.4%) |
| Current | 85 (17.4%) |
| CAGE (Problem Drinking) |  |
| No | 368 (75.4%) |
| Yes | 78 (16.0%) |
| NA | 42 (8.6%) |
| Physical Activity |  |
| Low | 139 (28.3%) |
| Moderate | 175 (35.9%) |
| Vigorous | 168 (34.4%) |
| NA | 6 (1.2%) |
| Diabetes Wave 1 | 37 (7.6%) |
| Hypertension Wave 1 | 200 (41.0%) |
| Depression Wave 1 | 41 (8.4%) |
| Frailty |  |
| Robust | 274 (56.6%) |
| Pre-Frail | 158 (32.4%) |
| Frail | 56 (11.5%) |
| Cardiovascular Disease Wave 1 | 82 (16.8%) |
| 12-year Mortality | 52 (10.7%) |
| Disability | 46 (9.4%) |
| Gait Speed | 136.2 (21.5) |
| Grip Strength | 26.6 (9.38) |
| MoCA Errors | 4.93 (3.34) |
| MMSE Errors | 1.52 (1.94) |

**Supplementary Table 3. HRS Cohort Characteristics.**

| **Characteristic** | **Mean (*SD*) or N (%)** |
| --- | --- |
| Age | 69.4 (9.6) |
| Sex Female | 2349 (58.5%) |
| Ethnicity |  |
| Non-Hispanic White | 2669 (66.4%) |
| Non-Hispanic Black | 657 (16.4%) |
| Hispanic/Latinx | 567 (14.1%) |
| Other | 122 (3.0%) |
| Education |  |
| Less than high school | 675(16.8%) |
| High school graduate | 1324 (33.0%) |
| Some college | 1051 (26.2%) |
| College and above | 967 (24.1%) |
| Education years | 12.8 (3.2) |
| BMI | 28.9 (6.3) |
| Smoking History |  |
| Never | 1851 (46.1%) |
| Former | 1775 (44.2%) |
| Current | 455 (11.3%) |
| Alcohol Intake |  |
| None | 2433 (60.6%) |
| Moderate | 1495 (37.2%) |
| Heavy | 72 (1.8%) |
| NA | 18 (0.4%) |
| Physical Activity |  |
| Light | 2206 (54.9%) |
| Moderate | 1986 (49.4%) |
| Vigorous | 991 (24.7%) |
| Diabetes | 1177 (29.1%) |
| Hypertension | 2578 (64.2%) |
| Depression | 585 (14.6%) |
| Frailty (Frailty Index) | 21.8 (14.7) |
| Cardiovascular Disease | 1298 (32.3%) |
| 6-year Mortality | 736 (18.3%) |
| Disability | 906 (22.5%) |
| Gait Speed | 79.9 (23.5) |
| Grip Strength | 30.4 (10.9) |
| Cognitive Errors | 5.3 (5.1) |


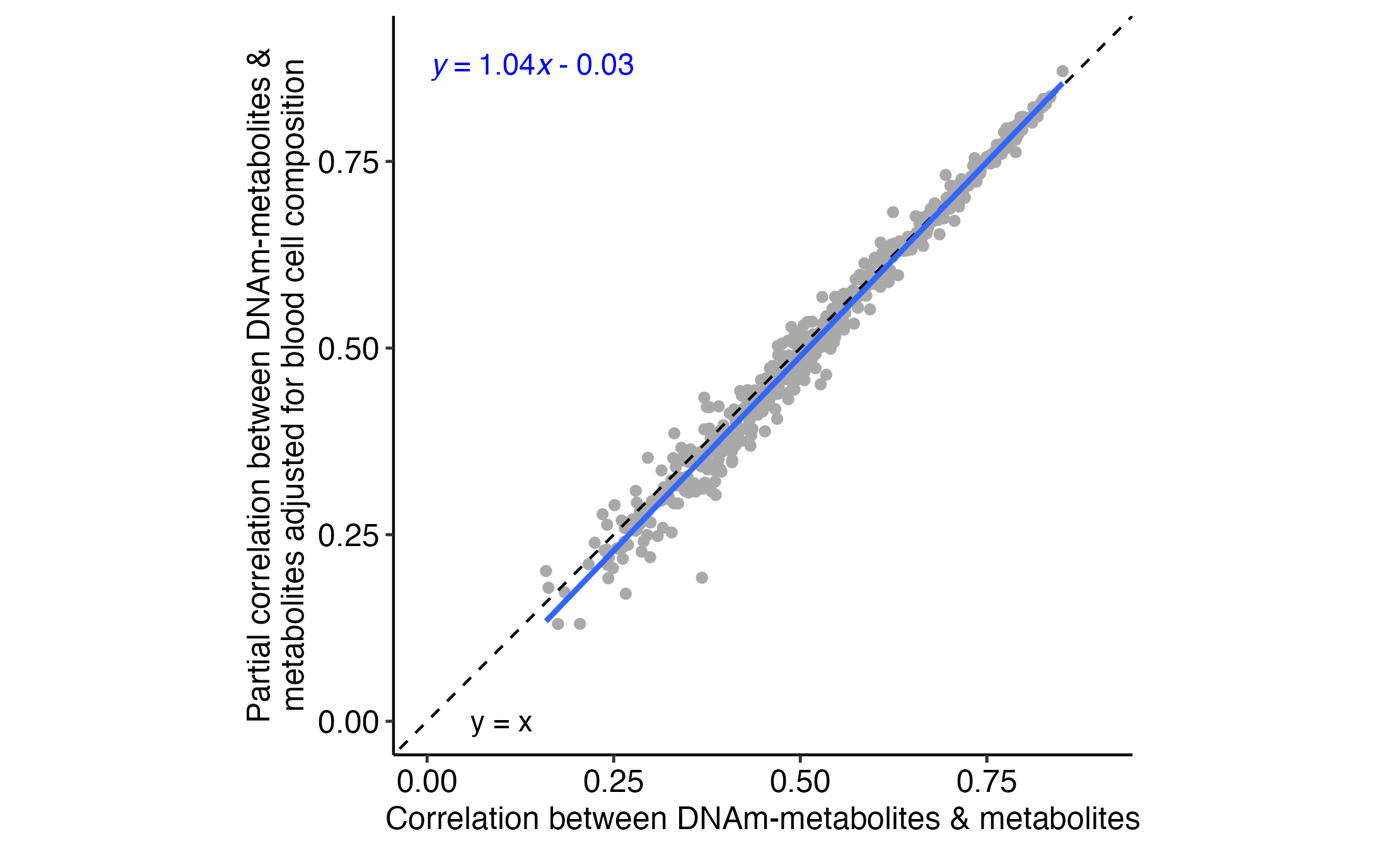


**Supplementary Figure 1. Pearson’s correlation between DNAm-metabolites and metabolites with and without adjustment for blood cell composition** (counts of naïve CD8+ T cells, memory and effector T cells, plasmablasts, CD4+ T cells, natural killer cells, monocytes, and granulocytes)**.**

**Supplementary Figure 2. DNAm-metabolic and metabolic age accelerations retained associations with most non-communicable disease risk factors after additional adjustment for BMI and smoking.** Age accelerations were scaled and linearly regressed on each risk factor (effect sizes shown with 95% confidence intervals), adjusting for age, sex, ethnicity, BMI, and smoking status (N = 820).

**Supplementary Figure 3. DNAm-metabolic age accelerations retained the directions of effects in health outcome predictions after additional adjustments for BMI and smoking in TILDA (N = 488) & HRS (N = 4018). (A)** Health outcomes were regressed on scaled age accelerations in TILDA (effect sizes shown with 95% confidence intervals), adjusting for age, sex, BMI, and smoking status, using Cox regression (time to death), logistic regression (cross-sectional diabetes, cardiovascular disease (CVD), disability, depression, pre-frailty/frailty), linear regression (gait speed, grip strength), and negative binomial regression (errors in the Montreal Cognitive Assessment (MoCA) and the Mini-Mental State Examination (MMSE)). (B) Health outcomes were regressed on scaled age accelerations in HRS, adjusting for age, sex, ethnicity, BMI, and smoking status, using Cox regression (time to death), logistic regression (diabetes, CVD, disability, depression), and linear regression (Frailty Index, gait speed, grip strength, cognitive error).

Age accelerations were scaled and linearly regressed on each risk factor (effect sizes shown with 95% confidence intervals), adjusting for age, sex, ethnicity, BMI, and smoking status (N = 820).

**Reference for Supplementary Information**

Aryee MJ, Jaffe AE, Corrada-Bravo H, Ladd-Acosta C, Feinberg AP, Hansen KD & Irizarry RA (2014) Minfi: A flexible and comprehensive Bioconductor package for the analysis of Infinium DNA methylation microarrays. *Bioinformatics* 30, 1363–1369. Available at: https://dx.doi.org/10.1093/bioinformatics/btu049 [Accessed November 6, 2024].

Chambers MC, MacLean B, Burke R, Amodei D, Ruderman DL, Neumann S, Gatto L, Fischer B, Pratt B, Egertson J, Hoff K, Kessner D, Tasman N, Shulman N, Frewen B, Baker TA, Brusniak MY, Paulse C, Creasy D, Flashner L, Kani K, Moulding C, Seymour SL, Nuwaysir LM, Lefebvre B, Kuhlmann F, Roark J, Rainer P, Detlev S, Hemenway T, Huhmer A, Langridge J, Connolly B, Chadick T, Holly K, Eckels J, Deutsch EW, Moritz RL, Katz JE, Agus DB, MacCoss M, Tabb DL & Mallick P (2012) A cross-platform toolkit for mass spectrometry and proteomics. *Nat. Biotechnol.* 30, 918–920. Available at: https://www.nature.com/articles/nbt.2377 [Accessed April 1, 2024].

Dona AC, Jiménez B, Schafer H, Humpfer E, Spraul M, Lewis MR, Pearce JTM, Holmes E, Lindon JC & Nicholson JK (2014) Precision high-throughput proton NMR spectroscopy of human urine, serum, and plasma for large-scale metabolic phenotyping. *Anal. Chem.* 86, 9887–9894. Available at: https://pubs.acs.org/doi/abs/10.1021/ac5025039 [Accessed April 1, 2024].

Faul JD, Kim JK, Levine ME, Thyagarajan B, Weir DR & Crimmins EM (2023) Epigenetic-based age acceleration in a representative sample of older Americans: Associations with aging-related morbidity and mortality. *Proc. Natl. Acad. Sci. U. S. A.* 120, e2215840120.

Folstein MF, Folstein SE & McHugh PR (1975) “Mini-mental state”. A practical method for grading the cognitive state of patients for the clinician. *J. Psychiatr. Res.* 12, 189–198.

Fong TG, Fearing MA, Jones RN, Shi P, Marcantonio ER, Rudolph JL, Yang FM, Kiely DK & Inouye SK (2009) Telephone Interview for Cognitive Status: Creating a crosswalk with the Mini-Mental State Examination. *Alzheimer’s Dement.* 5, 492–497.

Graf C (2008) The lawton instrumental activities of daily living scale. *Am. J. Nurs.* 108, 52–62. Available at: https://pubmed.ncbi.nlm.nih.gov/18367931/ [Accessed April 1, 2024].

Izzi-Engbeaya C, Comninos AN, Clarke SA, Jomard A, Yang L, Jones S, Abbara A, Narayanaswamy S, Eng PC, Papadopoulou D, Prague JK, Bech P, Godsland IF, Bassett P, Sands C, Camuzeaux S, Gomez-Romero M, Pearce JTM, Lewis MR, Holmes E, Nicholson JK, Tan T, Ratnasabapathy R, Hu M, Carrat G, Piemonti L, Bugliani M, Marchetti P, Johnson PR, Hughes SJ, James Shapiro AM, Rutter GA & Dhillo WS (2018) The effects of kisspeptin on β-cell function, serum metabolites and appetite in humans. *Diabetes, Obes. Metab.* 20, 2800–2810. Available at: /pmc/articles/PMC6282711/ [Accessed April 1, 2024].

Jiménez B, Holmes E, Heude C, Tolson RF, Harvey N, Lodge SL, Chetwynd AJ, Cannet C, Fang F, Pearce JTM, Lewis MR, Viant MR, Lindon JC, Spraul M, Schäfer H & Nicholson JK (2018) Quantitative Lipoprotein Subclass and Low Molecular Weight Metabolite Analysis in Human Serum and Plasma by 1H NMR Spectroscopy in a Multilaboratory Trial. *Anal. Chem.* 90, 11962–11971. Available at: https://doi.org/10.1021/acs.analchem.8b02412.

Katz S, Downs TD, Cash HR & Grotz RC (1970) Progress in development of the index of ADL. *Gerontologist* 10, 20–30. Available at: https://doi.org/10.1093/geront/10.1_Part_1.20.

Lewis MR, Chekmeneva E, Camuzeaux S, Sands CJ, Yuen AHY, David M, Salam A, Chappell K, Cooper B, Haggart GA, Maslen L, Gómez-Romero M, Horneffer-Van Der Sluis V, Correia G & Takats Z (2022) An Open Platform for Large Scale LC-MS-Based Metabolomics. *ChemRxiv*. Available at: https://chemrxiv.org/engage/chemrxiv/article-details/61ebd6fa0716a8529e3823dc [Accessed March 30, 2024].

Measures P & Arbor A (2008) HRS Documentation Report. *Blood Press.*, 1–18.

Mekli K, Stevens A, Marshall AD, Arpawong TE, Phillips DF, Tampubolon G, Lee J, Prescott CA, Nazroo JY & Pendleton N (2018) Frailty Index associates with GRIN2B in two representative samples from the United States and the United Kingdom. *PLoS One* 13, e0207824.

Nasreddine ZS, Phillips NA, Bédirian V, Charbonneau S, Whitehead V, Collin I, Cummings JL & Chertkow H (2005) The Montreal Cognitive Assessment, MoCA: A brief screening tool for mild cognitive impairment. *J. Am. Geriatr. Soc.* 53, 695–699.

Radloff LS (1977) The CES-D Scale: A Self-Report Depression Scale for Research in the General Population. *Appl. Psychol. Meas.* 1, 385–401.

Roe L, Normand C, Wren MA, Browne J & O’Halloran AM (2017) The impact of frailty on healthcare utilisation in Ireland: Evidence from the Irish longitudinal study on ageing. *BMC Geriatr.* 17, 203. Available at: https://doi.org/10.1186/s12877-017-0579-0.

Romero-Ortuno R (2013) An alternative method for Frailty Index cut-off points to define frailty categories. *Eur. Geriatr. Med.* 4, 299–303.

Sands CJ, Wolfer AM, Correia GDS, Sadawi N, Ahmed A, Jiménez B, Lewis MR, Glen RC, Nicholson JK & Pearce JTM (2019) The nPYc-Toolbox, a Python module for the pre-processing, quality-control and analysis of metabolic profiling datasets. *Bioinformatics* 35, 5359–5360. Available at: https://doi.org/10.1093/bioinformatics/btz566.

Smith CA, Want EJ, O’Maille G, Abagyan R & Siuzdak G (2006) XCMS: Processing mass spectrometry data for metabolite profiling using nonlinear peak alignment, matching, and identification. *Anal. Chem.* 78, 779–787. Available at: https://pubs.acs.org/doi/abs/10.1021/ac051437y [Accessed April 1, 2024].

Ward M, May P, Briggs R, McNicholas T, Normand C, Kenny RA & Nolan A (2020) Linking death registration and survey data: Procedures and cohort profile for The Irish Longitudinal Study on Ageing (TILDA). *HRB Open Res.* 3. Available at: /pmc/articles/PMC7376615/ [Accessed March 30, 2024].

Wolfer AM, Correia GDS, Sands CJ, Camuzeaux S, Yuen AHY, Chekmeneva E, Takats Z, Pearce JTM & Lewis MR (2021) Extraction and integration of annotated metabolic features in LC-MS profiling datasets. *Bioinformatics* 37, 4886–4888. Available at: https://academic.oup.com/bioinformatics/article/37/24/4886/6298587 [Accessed October 21, 2022].

Zivin K, Llewellyn DJ, Lang IA, Vijan S, Kabeto MU, Miller EM & Langa KM (2010) Depression among older adults in the United States and England. *Am. J. Geriatr. Psychiatry* 18, 1036–1044.
